# Separable Transition Density in the Hybrid Model for Tumor-Immune System Competition

**DOI:** 10.1155/2012/610124

**Published:** 2012-01-12

**Authors:** Carlo Cattani, Armando Ciancio

**Affiliations:** ^1^Department of Mathematics, University of Salerno, Via Ponte Don Melillo, 84084 Fisciano, Italy; ^2^Department of Mathematics, University of Messina, Viale Ferdinando Stagno d'Alcontres 31, 98166 Messina, Italy

## Abstract

A hybrid model, on the competition tumor cells immune system, is studied under suitable hypotheses. The explicit form for the equations is obtained in the case where the density function of transition is expressed as the product of separable functions. A concrete application is given starting from a modified Lotka-Volterra system of equations.

## 1. Introduction

The competition between tumor cells and the immune system is mainly due to a significant presence of the proliferation and/or destructive events. In particular, cancer cells have the ability of expressing their biological activity to escape from the immune system which, in principle, have to challenge the progressing cells. The biological activity is not generally the same for all cells since it is statically distributed. 

 Several authors [1–7] have applied the methods of the classical mathematical kinetic theory of gases to study the immune competition with special attention to cancer phenomena. In this approach, one has to take account of statistical averages and stochastic parameters, typical of macromodels. 

 Other authors [8–15] have proposed mathematical models based on nonlinear differential equations, which generalize the classical Lotka-Volterra equations. These equations, as known, follow from a deterministic approach on a microscale. 

 In some recent papers [16–20], a hybrid model was proposed which can be considered as an alternative method between the above two approaches, aiming to mix the two scales into a unique set of equations, the hybrid model. In this model, a system of nonlinear ordinary differential equations are coupled with a stochastic parameter generated by the (kinetic) interaction between the tumor cells and the immune system. 

 This time-depending stochastic parameter was linked [17] to the hiding-learning information process which underlies the cells competition. In particular [17], the hiding-learning dynamics appears between two populations (tumor cells-immune system) in which the first one has an uncontrolled proliferating and hiding ability and the second one has higher destructive ability and the need of learning about the presence of the first population.

 In this paper, we study the above hybrid model by assuming a particular form of the stochastic coefficient. There follow interesting results on the model and, moreover, the classical model of Lotka-Volterra modified by the hiding-learning process can be derived as a special case. 

## 2. Modelling the Immune Competition of Complex Systems

Let us consider a system of two interacting and competing populations. Each population is constituted by a large number of individuals called active particles; their microscopic state is called (biological) activity. This activity enables the particle to organize a suitable response with respect to any information process. In absence of prior information, the activity reduces either to a minimal loss of energy or to a random process.

In active particle competitions, the simplest model of binary interaction is based on proliferation-destructive competition. So that, when the first population get aware of the existence of the other challenging population, it starts to proliferate and destroy the competing cells. However, in this process the most important step is the ability of cells to hide themselves and to learn about the activity of the competing population.

In details consider a physical system of two interacting populations each one constituted by a large number of active particles with sizes:

(1) $$\;n_{i}=n_{i}\left(t\right),\;\left(n_{i}\left(t\right):\left[0,T\right]\to {\mathbb{R}}_{+};\;\;i=1,2\right).$$

Particles are homogeneously distributed in space, while each population is characterized by a microscopic state, called activity, denoted by the variable *u*. The physical meaning of the microscopic state may differ for each population. We assume that the competition model depends on the activity by a function of the overall distribution:

(2) $$\mu =\mu \left[f_{i}\left(t,u\right)\right],\;\left(\mu \left[f_{i}\left(t,u\right)\right]:{\mathbb{R}}_{+}\to {\mathbb{R}}_{+}\right).$$

The description of the overall distribution over the microscopic state within each populations is given by the probability density function:

(3) $$\;\begin{array}{c} f_{i}=f_{i}\left(t,u\right),\\ \left(f_{i}\left(t,u\right):\left[0,T\right]\times D_{u}\to {\mathbb{R}}_{+},\;\;D_{u}\subseteq \mathbb{R};\;\;i=1,2\right) \end{array}$$

such that *f*_*i*_(*t*, *u*)*du* is the probability that the activity *u* of particles of the *i*th population, at the time *t*, ranges in the interval [*u*, *u* + *du*]. 

 Moreover, it is

(4) ∀i, ∀t≥0:0≤fi(t,u)≤1, ∫Dufi(t,u)du=1.

We will see in Sections 3 and 4 how the microscopic structure influences the macroscopic system.

## 3. Hybrid Model

We consider, in this section, the competition between two cell populations: the first one with uncontrolled proliferating ability and with hiding ability; the second one with higher destructive ability, but with the need of learning about the presence of the first population. The analysis developed in what follows is referring to a specific case where the second population attempts to learn about the first population which, instead, escapes by modifying its appearance. Specifically, the hybrid evolution equations can be formally written as follows:

(5) $$\begin{array}{c} \frac{dn_{i}}{dt}=G_{i}\left(n_{1},n_{2};\mu \left[f\right]\right),\;\\ \frac{\partial f_{i}}{\partial t}={𝒜}_{i}\left[f\right], \end{array}$$

where

1. *G*_*i*_, for *i* = 1,2, is a function of *n* = {*n*_1_, *n*_2_},
2. *μ*, acts over *f* = {*f*_1_, *f*_2_},
3. *𝒜*_*i*_, for *i* = 1,2, is a nonlinear operator acting on *f*,
4. *μ*[*f*] is a functional (0 ≤ *μ* ≤ 1) which describes the ability of the second population to identify the first one.

As a consequence, (5) denotes a hybrid system of a deterministic system coupled with a microscopic system statistically described by a kinetic theory approach. In the following, the evolution of density distribution will be taken within the kinetic theory.

The derivation of (5)_2_ can be obtained starting from a detailed analysis of microscopic interactions. Specifically, consider binary interactions between a test, or candidate, particle with state *u*_∗_ belonging to the *i*th population, and field particle with state u* belonging to the *j*th population. We assume that microscopic interactions are characterized by the following quantities.

- The encounter rate, which depends, for each pair of interacting populations on a suitable average of the relative velocity *η*_*ij*_, with *i*, *j* = 1,2.
- The transition density function *φ*_*ij*_(*u*_∗_, u*, *u*), denotes the probability density that a candidate particle with activity *u*_∗_ belonging to the *i*th population, falls into the state *u* ∈ *D*_*u*_, of the test particle, after an interaction with a field entity, belonging to the *j*th population, with state u*. The probability density *φ*_*ij*_(*u*_∗_, u*, *u*) fulfills the condition
  (6) ∀i,j, ∀u∗,u∗:∫Duφij(u∗,u∗,u)du=1,         φij(u∗,u∗,u)>0.
  Then, by using the mathematical approach, developed in [17], it yields the following class of evolution equations:
  (7)   ∂fi∂t(t,u)=∑j=12∫Du×Duηijφij(u∗,u∗,u)     ×fi(t,u∗)fj(t,u∗)du∗du∗ −fi(t,u)∑j=12∫Duηijfj(t,u∗)du∗,
  which can be formally written as (5)_2_.

Since our model is based on the hiding-learning dynamics, one has to introduce the functional which takes into account the “distance” between the two distribution so that *μ* in (5) is defined as

(8) $$\mu \left[f_{i},f_{j}\right]\left(t\right)=\mu \left(\left|f_{i}-f_{j}\right|\right)\left(t\right)$$

with

(9) $$\begin{array}{c} 0\leq \mu \left[f_{i},f_{j}\right]\left(t\right)\leq 1,\;\forall u\in D_{u}\wedge t\in T,\\ \mu \left[f_{i},f_{j}\right]\left(t\right)=1\Leftrightarrow f_{i}=f_{j},\;\\ \mu \left[f_{i},f_{j}\right]\left(t\right)=0\Leftrightarrow f_{i}=0\vee f_{j}=0, \end{array}$$

where the maximum learning result is obtained when the second population is able to reproduce the distribution of the first one: *f*_1_ = *f*_2_, while the minimum learning is achieved when one distribution is vanishing.

In some recent papers [5–7, 17], it has been assumed that

(10) $$\mu \left[f_{i},f_{j}\right]\left(t\right)=\mu \left(\left|f_{i}-f_{j}\right|\right)\left(t\right)=1-\int_{D_{u}}{\left(f_{1}-f_{2}\right)}^{2}\left(t,u\right)du.$$

In this case, it is *μ* = 1, when *f*_1_ = *f*_2_, otherwise *μ* ≠ 1 with *μ* ↓ 0, depending on the time evolution of the distance between *f*_1_ and *f*_2_. There follows that this parameter could have an infinite value range.

Thus, we have

(11) $$\begin{array}{cc} & 0\leq \mu \left[f\right]\left(t\right)\leq 1\Rightarrow 0\leq \int_{D_{u}}{\left(f_{1}-f_{2}\right)}^{2}\left(t,u\right)du\leq 1,\\ & \;\;\;\;\;\;\;\;\;\;\;\;\;\;\;\;\forall t\in \left[0,T\right]. \end{array}$$

Notice that *μ* is the coupling term which links the macroscopic model (5)_1_ to the microscopic model (5)_2_.

## 4. Transition Density Function Based on Separable Functions

In order to find some classes of solutions of (7), we assume that the transition density is the product of separable density functions as

(12) $$\begin{array}{c} \varphi_{ij}\left(u_{*},u^{*},u\right)=\left(1-\delta_{ij}\right)\psi_{i}\left(u_{*},u\right)\xi_{j}\left(u^{*},u\right),\; \end{array}$$

that is,

(13) $$\begin{array}{c} \varphi_{11}=\varphi_{22}=0,\\ \varphi_{12}\left(u_{*},u^{*},u\right)=\psi_{1}\left(u_{*},u\right)\xi_{2}\left(u^{*},u\right),\\ \varphi_{21}\left(u_{*},u^{*},u\right)=\psi_{2}\left(u_{*},u\right)\xi_{1}\left(u^{*},u\right), \end{array}$$

and using (10) one has

(14) $$\begin{array}{c} \int_{D_{u}}\psi_{i}\left(u_{*},u\right)\xi_{j}\left(u^{*},u\right)du=1\;\left(i\neq j\right),\\ \psi_{i}\left(u_{*},u\right)>0,\;\;\xi_{j}\left(u^{*},u\right)>0\;\left(i,j=1,2\right). \end{array}$$

By a substitution of the above terms into (7) we get

(15) $$\begin{array}{cc} & \frac{\partial f_{1}}{\partial t}\left(t,u\right)\\ & \;=\overset{2}{\underset{j=1}{\sum }}\int_{D_{u}\times D_{u}}\eta_{1j}\varphi_{1j}\left(u_{*},u^{*},u\right)f_{1}\left(t,u_{*}\right)f_{j}\left(t,u^{*}\right)du_{*}du^{*}\\ & \;\;-f_{1}\left(t,u\right)\overset{2}{\underset{j=1}{\sum }}\int_{D_{u}}\eta_{1j}f_{j}\left(t,u^{*}\right)du^{*},\\ & \frac{\partial f_{2}}{\partial t}\left(t,u\right)\\ & \;=\overset{2}{\underset{j=1}{\sum }}\int_{D_{u}\times D_{u}}\eta_{2j}\varphi_{2j}\left(u_{*},u^{*},u\right)f_{2}\left(t,u_{*}\right)f_{j}\left(t,u^{*}\right)du_{*}du^{*}\\ & \;\;-f_{2}\left(t,u\right)\overset{2}{\underset{j=1}{\sum }}\int_{D_{u}}\eta_{2j}f_{j}\left(t,u^{*}\right)du^{*}, \end{array}$$

from where, by taking into account (13), we obtain

(16) $$\begin{array}{cc} & \frac{\partial f_{1}}{\partial t}\left(t,u\right)\\ & \;\;=\eta_{12}\int_{D_{u}}\psi_{1}\left(u_{*},u\right)f_{1}\left(t,u_{*}\right)du_{*}\int_{D_{u}}\xi_{2}\left(u^{*},u\right)f_{2}\left(t,u^{*}\right)du^{*}\\ & \;\;\;-f_{1}\left(t,u\right)\left[\eta_{11}\int_{D_{u}}f_{1}\left(t,u^{*}\right)du^{*}+\eta_{12}\int_{D_{u}}f_{2}\left(t,u^{*}\right)du^{*}\right],\\ & \frac{\partial f_{2}}{\partial t}\left(t,u\right)\\ & \;\;=\eta_{21}\int_{D_{u}}\psi_{2}\left(u_{*},u\right)f_{2}\left(t,u_{*}\right)du_{*}\int_{D_{u}}\xi_{1}\left(u^{*},u\right)f_{1}\left(t,u^{*}\right)du^{*},\\ & \;\;\;-f_{2}\left(t,u\right)\left[\eta_{21}\int_{D_{u}}f_{1}\left(t,u^{*}\right)du^{*}+\eta_{22}\int_{D_{u}}f_{2}\left(t,u^{*}\right)du^{*}\right].\; \end{array}$$

According to (4) and (13), we have the more general system for the transition density based on separable functions

(17) ∂f1∂t(t,u) =η12∫Duψ1(u∗,u)f1(t,u∗)du∗  ×∫Duξ2(u∗,u)f2(t,u∗)du∗−(η11+η12)f1(t,u),∂f2∂t(t,u) =η21∫Duψ2(u∗,u)f2(t,u∗)du∗  ×∫Duξ1(u∗,u)f1(t,u∗)du∗−(η21+η22)f2(t,u).

This system (17) can be solved when the two functions of (14)_2_ are given.

 As an example, let us solve this system under the following hypotheses:

(18) $$\begin{array}{c} \psi_{1}\left(u_{*},u\right)=\psi_{2}\left(u_{*},u\right)=\delta \left(u-u_{*}\right),\\ \xi_{1}\left(u^{*},u\right)=\xi_{2}\left(u^{*},u\right)=\delta \left(u-u^{*}\right), \end{array}$$

so that *ψ*_*i*_ and *ξ*_*j*_  (*i*, *j* = 1,2) are a Dirac-delta which fulfill (14)_1_

(19) $$\int_{D_{u}}\delta \left(u-u_{*}\right)\delta \left(u-u^{*}\right)du=\delta \left(u_{*}-u^{*}\right).$$

The system (17), by using (18), becomes

(20) $$\begin{array}{c} \frac{\partial f_{1}}{\partial t}\left(t,u\right)=\eta_{12}\;f_{1}\left(t,u\right)f_{2}\left(t,u\right)-\left(\eta_{11}+\eta_{12}\right)f_{1}\left(t,u\right),\\ \frac{\partial f_{2}}{\partial t}\left(t,u\right)=\eta_{21}\;f_{1}\left(t,u\right)f_{2}\left(t,u\right)-\left(\eta_{21}+\eta_{22}\right)f_{2}\left(t,u\right). \end{array}$$

Moreover, by assuming that

(21) $$\eta_{11}=\eta_{21}=\eta_{11}=\eta_{22}\overset{\mathrm{def}}{=}\eta ,$$

and putting

(22) $$\;f\left(t,u\right)=f_{1}\left(t,u\right)-f_{2}\left(t,u\right),$$

from (20), one has

(23) ∂f(t,u)∂t=−2ηf(t,u).

The more general solution of this equation is

(24) $$f\left(t,u\right)=f\left(0,u\right)e^{-2\eta t}.$$

Assuming that

(25) $$\begin{array}{c} f\left(0,u\right)=\frac{1}{\sqrt{\pi }}e^{-u^{2}}, \end{array}$$

equation (24) becomes

(26) f(t,u)=1πe−(u2  +  2ηt).

From (10), by virtue of (22) and (26), we have

(27) $$\mu \left(t\right)=1-\int_{D_{u}}\frac{1}{\pi }e^{-2(u^{2}+2\eta t)}du.$$

Taking into account that

(28) $$\overset{+\infty }{\underset{-\infty }{\int }}\frac{1}{\pi }e^{-2(u^{2}+2\eta t)}du=\frac{e^{-4\eta t}}{\sqrt{2\pi }},$$

equation (27) gives

(29)   μ(t)=1−e−4ηt2π.

## 5. A Simple Application

It is well known that the pioneering Lotka-Volterra's model of two interacting and competing populations (*x* = prey, *y* = predatory) is based on the following differential system:

(30) $$\begin{array}{c} \frac{dx}{dt}=ax-bxy,\;\\ \frac{dy}{dt}=cxy-dy, \end{array}$$

where *a*, *b*, *c*, and *d* are constants. 

**Figure 1:**
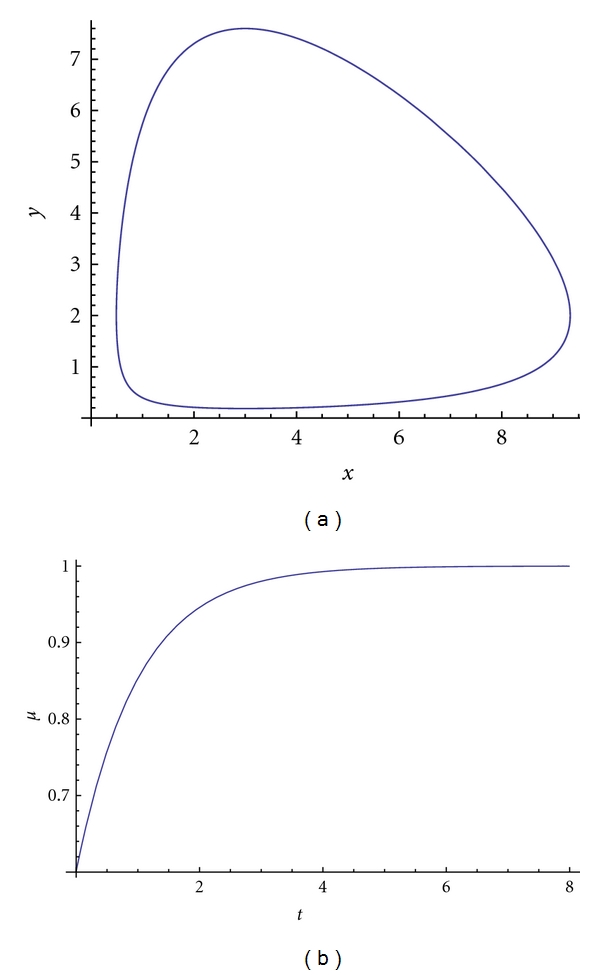
Time evolutions of the orbits of (30) with parameters *a* = 2, *b* = *c* = 1, and *d* = 3 (a); distribution function *μ*(*t*) for *μ* = 1/4 (b).

 In this model, the hiding-learning processes are not considered and the interaction and competition of the two populations start immediately. The orbits of the solutions of (30) are circles around the equilibrium point: *x* = *d*/*c*, *y* = *a*/*b* (see Figure 1). 

**Figure 2:**
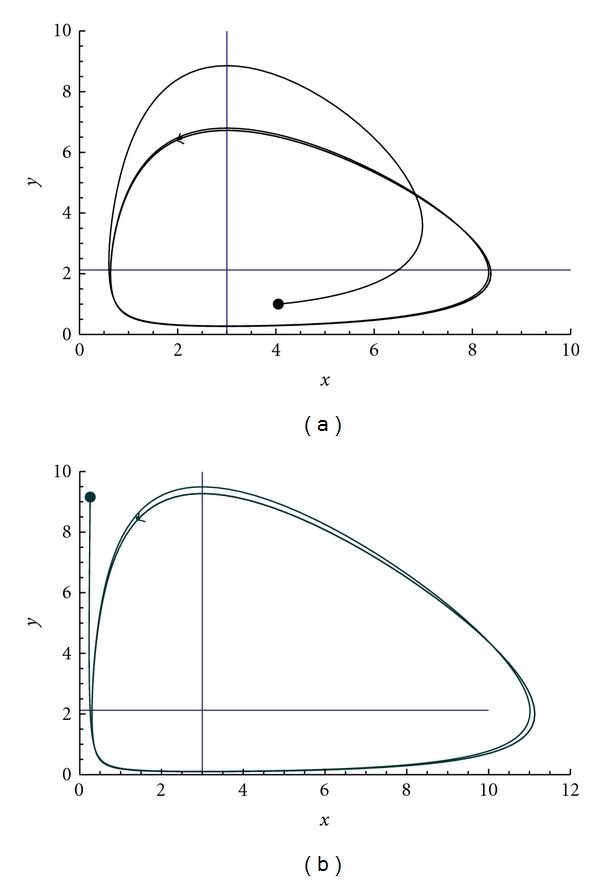
Time evolutions of the orbits of (32) with parameters *a* = 2, *b* = *c* = 1, and *d* = 3 and initial populations *x*_0_ = 4, *y*_0_ = 1 (a), *x*_0_ = 0.1, *y*_0_ = 9 (b).

If the hiding-learning processes occur, by using the results discussed in the previous sections, we propose the following system:

(31) $$\begin{array}{c} \frac{dx}{dt}=ax-\mu bxy,\;\\ \frac{dy}{dt}=cxy-dy,\; \end{array}$$

where *μ*, given by (29), is the functional (stochastic) parameter depending on the distribution of populations (see Figure 2). 

 The system (31) becomes

(32) $$\begin{array}{c} \frac{dx}{dt}=ax-b\left(1-\frac{e^{-4\eta t}}{\sqrt{2\pi }}\right)xy,\;\\ \frac{dy}{dt}=cxy-dy. \end{array}$$

The nonzero equilibrium point is

(33) $$\begin{array}{c} x=\frac{d}{c},\;\;y=\frac{a}{b\;\left(1-\left(e^{-4\eta t}/\sqrt{2\pi }\right)\right)}, \end{array}$$

where

(34) $$\underset{t\to \infty }{\lim}\frac{a}{b\;\left(1-\left(e^{-4\eta t}/\sqrt{2\pi }\right)\right)}=\frac{a}{b}.$$

For *η* = 1/4, the solutions of the system (32) are shown in Figure 2. 

 From Figure 2 it can be noticed that *x*_0_ > *y*_0_ so that the hiding-learning process delay the achievement of the circle around the nonzero equilibrium point. If *x*_0_ ≪ *y*_0_, then the cricle is reached more quickly.

## 6. Conclusion

In this paper, it has been studied a hybrid system of competition tumor cells versus immune system, within the kinetic model. A stochastic parameters is computed explicitly in the case of special transition density functions. A simple application shows that due to this parameters we obtain some more realistic solutions of the Lotka-Volterra system, where the cicle around the nonzero equilibrium point is shifted in time, thus showing the importance of the stochastic parameters in a correct approach to the analysis of competition models.
